# An Uncommon Encounter: Metastatic Olfactory Neuroblastoma in an Adult Male

**DOI:** 10.7759/cureus.73105

**Published:** 2024-11-06

**Authors:** Areti Kalfoutzou, Asimina Restemi, Cleopatra Rapti, Nikolaos Chaleplidis, Eleftheria Bagiokou, Dimitra Bartzi, Vasileios Ramfidis

**Affiliations:** 1 Department of Medical Oncology, 251 Air Force General Hospital, Athens, GRC; 2 Department of Pathology, 251 Air Force General Hospital, Athens, GRC

**Keywords:** chemotherapy, esthesioneuroblastoma, molecular testing, small round blue cell tumor, s: olfactory neuroblastoma

## Abstract

Olfactory neuroblastoma is an extremely rare malignancy of the sinonasal tract. It is a neuroectodermal tumor, originating from the olfactory epithelium of the nasal and sinus cavities. Due to its rarity, no standard of care treatments have been established to date. We present a case involving a young adult who presented for evaluation of a palpable cervical lymph node. Nasal endoscopy and Magnetic Resonance Imaging (MRI) of the visceral cranium showed a space-occupying lesion in the right nasal cavity, along with a suspicious right submandibular lymph node block. Fine Needle Biopsy (FNB) of the cervical lymph node was suggestive of a small round blue cell tumor, confirming the diagnosis of olfactory neuroblastoma. Staging Fluorodeoxyglucose Positron Emission Tomography/Computed Tomography (FDG-PET/CT) scan identified multiple bone-lytic metastases. The patient was treated with three lines of systemic therapy, but unfortunately, he succumbed to his disease. This case aims to highlight the intricacies of diagnosing and treating one of the rarest malignancies of the head and neck, particularly in the metastatic setting.

## Introduction

Olfactory neuroblastoma (ONB), or esthesioneuroblastoma, is a tumor originating from the olfactory neuroepithelium, accounting for 2-3% of the tumors of the sinonasal tract [1]. It is a neuroendocrine tumor with a variable clinical course and the potential for aggressive behavior and distant metastases [2]. Characterized by a wide range of clinical presentations, ONB can manifest symptoms associated with nasal obstruction, epistaxis, headaches, visual defects, and neurological complications, depending on tumor location and size [2]. This report aims to elucidate the clinical features, diagnostic challenges, and therapeutic strategies for this extremely rare tumor, emphasizing the importance of multidisciplinary management and the need for tailored treatment strategies in advanced-stage disease.

## Case presentation

A 35-year-old male, a fighter pilot by profession, presented for assessment of a self-detected enlarged lymph node in the right submandibular region. He reported no history of smoking and had an unremarkable past medical history. Upon palpation, the lymph node was hard, non-tender, and immobile. The patient reported no pain and was otherwise asymptomatic at presentation. Neurologic examination was insignificant, with normal tendon reflexes and muscle strength of 5/5 in both upper and lower extremities. Laboratory examinations were within normal limits.

**Figure 1:**
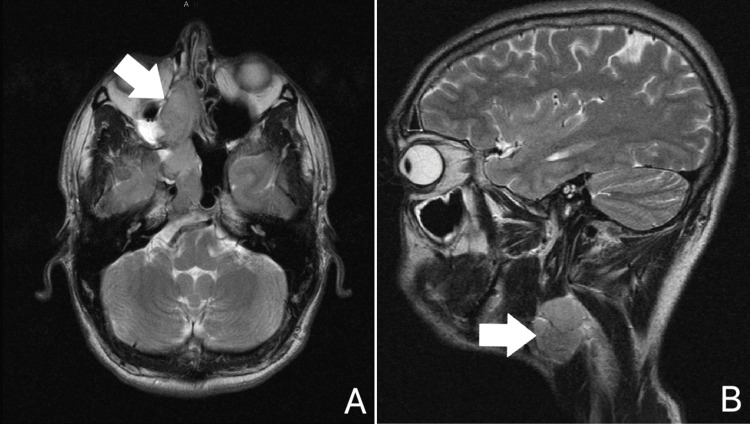
Axial MRI scan of the brain with intravenous contrast. Axial MRI scan of the brain with intravenous contrast demonstrating a lesion measuring 55x26x46 mm occupying the right nasal cavity (white arrow - panel A). Sagittal view displaying a right submandibular lymph node block (white arrow – panel B).

**Figure 2:**
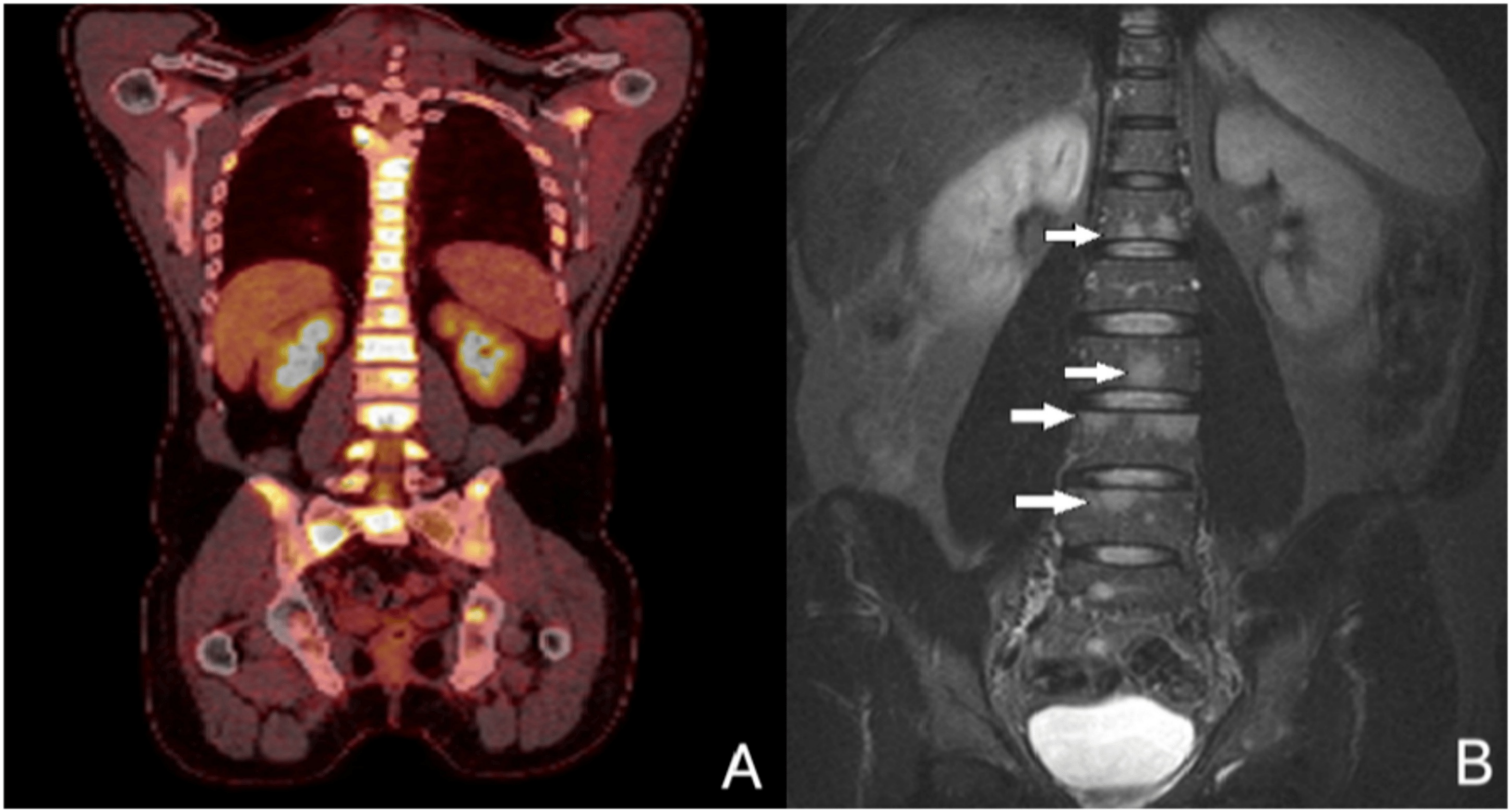
FDG-PET/CT scan. FDG-PET (Fluorodeoxyglucose Positron Emission Tomography) CT scan displaying multiple hypermetabolic bone lesions throughout the spine, in both scapulae and iliac bones (panel A). Coronal MRI scan of the spine demonstrating multiple metastatic bone-lytic lesions, exhibiting a high signal intensity in T2-weighed images (white arrows – panel B).

A neck ultrasound revealed a suspicious lymph node block in the right submandibular area. Magnetic Resonance Imaging (MRI) scan of the visceral cranium with intravenous contrast demonstrated a lesion measuring 55x26x46 mm, occupying the middle and upper right nasal cavity (Figure 1A). This lesion infiltrated the base of the skull at the level of the cribriform plate of the ethmoid bone, was in close contact with the right medial rectus muscle, and exhibited a low signal intensity on T1 and appeared isointense to the grey matter on T2-weighted images, next to a hyperintense area suggestive of fluid retention. Additionally, an enlarged lymph node measuring 45 mm with central necrosis was observed (Figure 1B). The right nasal cavity lesion and the lymph node displayed hypermetabolic activity on staging Fluorodeoxyglucose Positron Emission Tomography (FDG-PET) CT scan (SUVmax: 8.7, and 6.7 respectively). Bone-lytic lesions indicative of metastases along the spine and in the iliac bones were also apparent in both the FDG-PET/CT scan (Figure 2A) and the MRI scan of the spine (Figure 2B).

**Figure 3:**
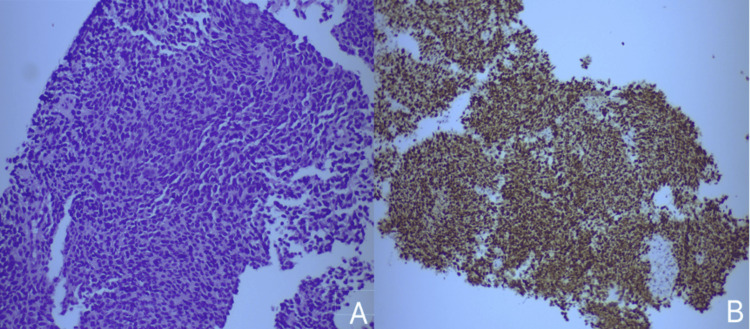
Histopathological examination. Histopathological examination revealed small, round cells characterized by an increased nuclear-to-cytoplasmic ratio, and basophilic, subround nuclei, indicative of a small round blue cell tumor (panel A, x20 magnification). The tumor cells exhibited strong positive staining for INSM1, a marker highly specific for neuroendocrine differentiation (panel B, x10 magnification).

The patient underwent a core biopsy of the cervical lymph node, and histopathological examination revealed small-sized cells with a high nuclear-to-cytoplasmic ratio and basophilic round nuclei, indicative of a small round blue cell tumor (Figure 3A). These nuclei lacked visible nucleoli and showed no signs of significant nuclear fusion, but there was a notably high mitotic activity, as evidenced by a ki-67 proliferation index of 85%. The cells were primarily arranged in a diffuse pattern, with some areas forming rosette-like structures, consistent with a Hyams grade III olfactory neuroblastoma. Further immunohistochemical testing showed positive staining for the following markers: INSM1 (Figure 3B), synaptophysin, keratin 8/18 (regionally), keratin AE1/AE3, p40 (focally), desmin (regionally), and myogenin. Negative immunostaining results were obtained for SOX-10, TTF-1, Chromogranin, S-100, and Calretinin.

Next-Generation Sequencing (NGS) analysis of the tumor tissue did not identify any actionable mutations. The results included a TP53 Exon 6 c.637C>T (p.R213) mutation, a retinoblastoma (RB1) c.1422-2A>G splicing mutation, Microsatellite Stability (MSS), a Tumor Mutational Burden (TMB) of 3.84 mutations per megabase, and a high Loss of Heterozygosity (LOH) at 34.48%.

The tumor was classified as stage D according to the Kadish system and as T2N1M1 according to the Dulguerov system. The case was reviewed by a Multidisciplinary Tumor Board (MDT), which determined the tumor to be unresectable, and initiation of systemic therapy was decided. The patient began first-line treatment with cisplatin, etoposide, and durvalumab, along with zoledronic acid. He tolerated the treatment well, except for mild episodes of epistaxis. Subsequently, he developed symptoms including loss of smell, headaches, and back pain, which required high doses of opioid analgesics. After three cycles of therapy, restaging with visceral cranium MRI and FDG-PET/CT scans were conducted. These scans revealed increased avidity in the primary nasal lesion, the cervical lymph nodes, and the bone-lytic lesions, as well as a hypermetabolic lung nodule (SUVmax: 4.3), indicating disease progression.

The patient began second-line treatment with docetaxel and irinotecan, based on a retrospective analysis by Kiyota et al. [3]. He tolerated therapy well, apart from an episode of significant epistaxis that required hospital admission, and was managed with nasal packing and tranexamic acid. After two cycles of treatment, the patient reported increased pain in his right hip and difficulty moving his right leg and abducting his left arm. Electromyoneurography (EMG/ENG) revealed radicular neuropathy involving the C5 level in the cervical spine and the L4-L5 levels in the lumbar spine. Restaging MRI scans of the brachial plexus, spine, and pelvis showed new bone-lytic metastases involving both scapulae and femurs.

After exhausting most therapeutic options, a thorough literature review guided the decision to conduct a Technetium-99m - Octreotide scan, aiming to evaluate the potential for treatment with somatostatin analogs. The scan revealed a mild uptake of somatostatin receptors (SSTR2, SSTR3, SSTR5) within the right nasal cavity lesion, regrettably ruling out this treatment option.

The patient was subsequently referred to a specialized center for potential enrollment in a clinical trial. He received one cycle of temozolomide therapy while awaiting confirmation of enrollment. Unfortunately, he developed sudden paraplegia due to spinal cord compression, unresponsive to the prompt initiation of radiotherapy. Further workup indicated bone marrow infiltration. The patient's health rapidly declined, sadly leading to his demise one month following his referral.

## Discussion

Olfactory neuroblastoma (ONB), also known as esthesioneuroblastoma, is a rare neuroectodermal tumor arising from the olfactory epithelium in the nasal cavity and paranasal sinuses [1]. It has an annual incidence of 0.4 to 1 case per 100,000 people worldwide [4-7]. ONB shows a bimodal age distribution, primarily affecting children and adults in their fifth and sixth decades of life, and exhibits no gender predilection [7,8]. To date, no specific inherited or environmental factors have been conclusively linked to the development of this malignancy [7,9].

The clinical presentation is atypical and varies depending on tumor size and location, potentially causing central nervous system (CNS) symptoms such as headaches, dizziness, and seizures; nasal symptoms like epistaxis and anosmia; or ocular symptoms including proptosis, lacrimation, and visual defects [9-11]. Site-specific symptoms may also present according to the location of metastases, and paraneoplastic syndromes such as Syndrome of Inappropriate Antidiuretic Hormone (SIADH) or Cushing Syndrome have been scarcely reported in the literature [9,12]. Our case initially presented with palpable lymph node metastases and later over the course of the disease, exhibited epistaxis, headaches, and neurological symptoms due to bone-lytic metastases.

Imaging modalities such as CT and MRI scans are crucial for the initial evaluation of ONB, particularly for assessing its proximity to the orbital fossa or skull bones [13]. MRI is slightly superior; ONB typically appears hypointense to grey matter on T1-weighted images and isointense or hyperintense on T2-weighted images, with a homogenous contrast enhancement [13]. This enhancement may be more pronounced in areas of hemorrhage or retention [2,13]. A bone scan is necessary for initial staging due to the high incidence of asymptomatic bone metastases [9]. FDG-PET/CT scan is also highly effective in identifying distant metastases and monitoring response to treatment [13]. Additionally, 99mTc-Octreotide, 111In-Octreotide scan, or Gallium-68 DOTATOC PET may be useful to assess somatostatin receptor expression and guide treatment decisions regarding somatostatin-targeted therapies [10,12]. In our case, on MRI, the tumor appeared hypointense to the grey matter on T1 and isointense on T2-weighed images. Furthermore, it exhibited significant hypermetabolic activity on FDG-PET, as well as a mild uptake of somatostatin receptors on Technetium-99m - Octreotide scan.

Definitive diagnosis relies on histopathological examination, which reveals small round blue cells with a salt-and-pepper chromatin distribution, a high nuclear-to-cytoplasm ratio, basophilic nuclei, and scant cytoplasm [8,9]. The Hyams grading incorporates a variety of histopathological features, including mitotic activity, cellular architecture, and pleomorphism, as well as the presence of necrosis, calcification, gland proliferation, and neurofibrillary matrix or rosettes, to classify ONB as low-grade Hyams (LGH; Hyams I-II) and high-grade Hyams (HGH; Hyams III-IV) [1,14,15]. Immunohistochemistry typically shows positive staining for neuron-specific enolase (NSE), synaptophysin, chromogranin, ISNM-1, CD56, neurofilament protein (NP), and negative staining for markers of epithelial malignancies, melanoma, lymphoma, or Ewing sarcoma [9,10]. Conversely, the sustentacular cells surrounding the tumor commonly stain positive for S-100 and glial fibrillary acidic protein [9]. Proliferation marker Ki67 is usually high ranging from 10 to 50%, indicating an aggressive biologic behavior [2,12].

Due to the lack of treatment options, molecular analysis has become crucial for understanding the biological behavior of olfactory neuroblastoma (ONB). Among the gene alterations commonly discovered are those involving TP53, RET, EGFR, cMET, CDKN2A, PIK3CA, CDH1, NF1, PDGFRA [4]. Chromosomal alterations in chromosomes 3, 11, and 17 have also been identified in ONB patients [4]. However, none of these alterations has emerged as a potential therapeutic pathway. In our case, no targetable mutations were detected. 

ONB can expand intracranially to invade adjacent anatomical structures or metastasize to lymph nodes and distant organs such as the bones, lungs, and liver [10,11,16,17]. Staging of ONB follows the modified Kadish system, which classifies the disease into stages A, B, and C, and was recently updated to include stage D [5]. The Kadish staging system for ONB classifies them as follows: Kadish A indicates a tumor limited to the nasal cavity; Kadish B denotes extension into the paranasal sinuses; Kadish C signifies extension to the cribriform plate, skull base, orbit, or intracranial cavity; and Kadish D indicates the presence of cervical lymph node or distant metastases [5]. Additionally, the Dulguerov staging system, based on the TNM (tumor, node, metastasis) classification, provides a more detailed understanding of the tumor extent, nodal involvement, and the presence of metastases [5,7,9,13].

Due to the rare incidence of olfactory neuroblastoma (ONB), specific treatment guidelines are not well-established [7,9]. Surgical resection followed by radiotherapy is the mainstay of treatment for localized disease [5,7,11]. Surgical approaches have evolved from open to endoscopic techniques, which are associated with lower complication and morbidity rates [12]. Radiotherapy is critical in the perioperative management of both early and locally advanced ONB, whereas chemotherapy maintains a well-established role in the treatment of advanced-stage disease [1,18]. Commonly used chemotherapy regimens include cisplatin-etoposide, with or without doxorubicin, ifosfamide, and vincristine, or the etoposide, ifosfamide, and cisplatin (VIP) regimen (2,8,17). Targeted therapies such as pazopanib, sunitinib, and everolimus have been explored with varying outcomes, while the potential role of immunotherapy remains under-investigated [12]. Furthermore, treatment with radiopharmaceuticals like 177Lu-DOTATATE or 111In-octreotide has been explored in cases exhibiting somatostatin receptor expression [19].

The 10-year survival rate for olfactory neuroblastoma (ONB) is generally more favorable compared to other sinonasal tumors and varies significantly according to the stage, ranging from 83.4% in Kadish stage A to 13.3% in stage D [2,12]. The recurrence rate for ONB ranges between 30% and 60%, with recurrences typically occurring between 5 to 15 years post-treatment, highlighting the necessity for long-term follow-up. Critical prognostic factors that influence survival include the Hyams grade, the extent of tumor resection, and lymph node status [7,12,14,15,20]. These factors underscore the importance of comprehensive surgical management and diligent post-treatment monitoring in improving long-term outcomes for patients with ONB.

## Conclusions

Olfactory neuroblastoma (ONB) is a notably rare malignancy with a poor prognosis in advanced stages. The clinical presentation and unique location of the tumor should raise clinical suspicion for timely diagnosis. Optimal management of ONB necessitates a multidisciplinary approach, integrating the expertise of oncologists, pathologists, radiation oncologists, otolaryngologists, and neurosurgeons. Further exploration in the genomic field is necessary to advance treatment options. Furthermore, the rarity of this malignancy underscores the critical need for the establishment of an international case database and the promotion of rigorous clinical trials to enhance understanding and improve patient outcomes.
